# Stressed, Lonely, and Overcommitted: Predictors of Lawyer Suicide Risk

**DOI:** 10.3390/healthcare11040536

**Published:** 2023-02-11

**Authors:** Patrick R. Krill, Hannah M. Thomas, Meaghyn R. Kramer, Nikki Degeneffe, Justin J. Anker

**Affiliations:** 1 Krill Strategies, LLC, Minneapolis, MN 55408, USA; 2 Department of Psychiatry, University of Minnesota, Minneapolis, MN 55454, USA

**Keywords:** lawyers, suicidal ideation, occupational stress, loneliness, perceived stress, depression, mental health, work overcommitment

## Abstract

Suicide is a significant public health concern, and lawyers have been shown to have an elevated risk for contemplating it. In this study, we sought to identify predictors of suicidal ideation in a sample consisting of 1962 randomly selected lawyers. Using logistic regression analysis, we found that high levels of work overcommitment, high levels of perceived stress, loneliness as measured by the UCLA loneliness scale, and being male were all significantly associated with an increased risk of suicidal ideation. These results suggest that interventions aimed at reducing work overcommitment, stress, and loneliness, and addressing gender-specific risk factors, may be effective in reducing the risk of suicidal ideation among lawyers. Further research is needed to expand upon these findings and to develop and test interventions specifically tailored to the needs of this population.

## 1. Introduction

Lawyers contemplate suicide (suicidal ideation) at an exceedingly high rate. Suicidal ideation, defined as thoughts, ideas, or ruminations about ending one’s own life, is the first step to suicide and is predictive of suicide attempts [1,2]. Prior estimates suggest that between 10 and 12 percent of lawyers in the U.S. have contemplated suicide [3,4,5], compared to 4.2% of adults ≥ 18 years of age in U.S. population [6]. Given the high rates of suicidal ideation among lawyers, it is crucial to identify factors that potentially contribute to their suicide risk. 

Lawyers are prone to mental health issues, including anxiety, depression, and substance abuse [3,7], which are strongly linked to suicide risk [8,9,10,11,12]. A nationwide study of ~13,000 lawyers indicated that 28% experienced depression, 19% reported anxiety, 21% had alcohol use problems, and 11% had problems with drug use [3]. Lawyers also experience elevated levels of stress (i.e., perceiving events in one’s life or work as unpredictable, uncontrollable, and/or overloaded) [13,14] and loneliness (perceiving one’s social needs as not being met) [15,16,17] which are well-established predictors of suicide risk [18,19,20,21,22,23,24]. However, the relative contribution of lawyer mental health, stress, and loneliness to suicide risk has yet to be examined. 

Work-related hazards specific to the legal profession may also contribute to suicide risk. For example, lawyers are expected to work long hours, meet tight deadlines, and handle complex legal issues, all while maintaining a high level of professionalism and client satisfaction [5,13,25,26]. This can lead to burnout and feelings of being overwhelmed, which have been linked to increased risk of suicidal ideation [27,28,29,30,31,32,33,34,35]. Findings from other research, however, demonstrate that the association between job burnout and suicidal ideation disappears after adjusting for depression [36]. This highlights the importance of accounting for psychological distress when seeking to identify workplace predictors of suicidality.

Work-family conflict, or difficulty balancing work and family responsibilities, is a common stressor that can negatively impact mental health [37,38,39,40] and there is a growing body of research indicating that work-family conflict is a predictor of suicidal ideation [41,42]. Anker and Krill found that work-family conflict among lawyers was significantly associated with perceived stress and attrition due to burnout in a large sample of lawyers. These findings suggest that work-family conflict may also play a role in suicidal ideation among lawyers.

According to the World Health Organization, men are three times more likely than women to die by suicide even though women tend to experience higher levels of suicidal ideation [43]. Gender differences in suicide risk factors have also been observed across a range of occupational groups [30,44,45,46]. In relation to lawyers specifically, Anker and Krill (2021) [7] found that women lawyers were more likely to experience moderate to severe levels of work–family conflict, work overcommitment, perceived stress, anxiety, depression, and risky or hazardous levels of alcohol use compared to male lawyers. Owing to their higher prevalence of suicidality risk factors, we hypothesized that women lawyers may be at a higher risk for suicidal ideation than men.

Considering how many lawyers contemplate suicide and the paucity of data examining the relationship between their suicidal ideation and the known risk factors they often experience, further research on the subject is an overdue and essential step in the development of effective suicide prevention strategies tailored to that population. As such, the current study examined the relationship between suicidal ideation, and factors that negatively and disproportionally affect lawyers, including perceived stress, loneliness, work overcommitment, work-family conflict, alcohol use, and prior mental health diagnosis. 

## 2. Materials and Methods

### 2.1. Participants

#### Recruitment and Random Selection

The University of Minnesota Institutional Review Board reviewed the study design and protocol. Recruitment was coordinated in collaboration with the California Lawyers Association (“CLA”), a nonprofit, voluntary organization that includes the Sections of the State Bar of California and the California Young Lawyers Association, and the D.C. Bar, the largest unified bar in the United States and an organization which provides an oversight structure to maintain ethical standards and Rules of Professional Conduct. An advertisement was included in newsletters sent by the D.C. Bar and CLA to their respective member lists and posted on their organization’s website. The advertisement provided a summary of the study, indicating that the survey was anonymous and that members would be randomly invited to participate in the study via email. Participants were randomly selected from a list of unique de-identified I.D.s supplied by the CLA and D.C. Bar. Each list contained approximately 98,000 IDs (196,000 total IDs). Hence, 40,000 IDs were randomly selected from each list (80,000 total) using the random sample function in the statistical platform R. From that sample, 5292 participants consented to the survey and about 4000 completed the survey. An email notification was sent to randomly selected D.C. Bar and CLA members on behalf of the researchers. Seven days following the email notification, study candidates received an email containing a link to a REDCap (Research Electronic Data Capture) survey. Clicking on the link directed participants to the survey’s informed consent page. The study was conducted during the summer of 2020. 

### 2.2. Materials

#### 2.2.1. Descriptive Variables

Demographics and work context. Information regarding age, race, relationship status, and whether respondents had children was collected. Additionally, information on the following work-related variables was collected from participants: the average number of hours worked per week, current position in the legal profession, and whether the current position involved litigation.

#### 2.2.2. Measures

Mental Health Diagnoses. Participants were asked if they ever (lifetime) or currently (past 12 months) had a diagnosis of major depression, anxiety disorder, PTSD, bipolar disorder, alcohol use disorder, substance use disorder, or a non-specified mental health disorder.

Depression. Participants completed the Patient Health Questionnaire-9 (PHQ-9) to assess the prevalence and severity of symptoms of depression [47]. For the PHQ-9, participant depression severity scores were grouped across the following 5 categories: None/Minimal (0–4), Mild (5–9), Moderate (10–14), Moderately Severe (15–19), and Severe (20–27).

Stress. The total score on the Perceived Stress Scale (PSS) was used to assess how unpredictable, uncontrollable, and overloaded respondents found their lives [48]. Scores on the PSS were grouped into Low (0–13), Moderate (14–26), and Severe (27–40) categories for analyses comparing. 

Alcohol Use Severity. Scores on the Alcohol Use Disorders Identification Test (AUDIT-C) were used to assess risky drinking (women ≥ 3; men ≥ 4) and high-risk/hazardous drinking (women ≥ 4; men ≥ 5) [49].

Substance Use Severity. Scores on the DAST were used to assess substance use severity and were classified into the following four severity groups: Lifetime abstinence, No problems reported, Low, and Moderate to Severe [50].

Loneliness. Participants completed a 3-item questionnaire adapted from the Revised University of California, Los Angeles (UCLA) Loneliness Scale to assess the prevalence and severity of loneliness [51]. The questionnaire consisted of the following 3 items: “How often do you feel that you lack companionship?”, “How often do you feel left out?”, and “How often do you feel isolated from others?”. Participants responded with “hardly ever or never”, “some of the time”, and “often”. Ratings were summed to produce a loneliness score ranging from 3 to 9, with a higher score indicating greater loneliness. Following methods by Steptoe et al., (2013) [52], participants scores were summed and grouped across 2 categories (Lonely (3–5) and Not Lonely (6–9). 

Work Overcommitment. We used the overcommitment subscale of the Effort–Reward Imbalance (ERI) Questionnaire [53] to assess feelings of being overwhelmed by work demands. Responses on the subscale were on a four-point Likert scale (1 = Strongly Disagree, 2 = Disagree, 3 = Agree, 4 = Strongly Agree). 

Work-Family Conflict. The degree to which work interfered with family life was assessed using three items from the Work-Family Conflict (WFC) subscale from the short version of the Copenhagen Psychosocial Questionnaire [54]. Participants rated items are 4-point Likert-scale ranging from 1 (no, not at all) to 4 (yes, certainly). 

#### 2.2.3. Outcome Variables

Suicidality/Suicidal Ideation. Participants were classified as endorsing suicidality according to item 9 of the PHQ-9, which can accurately identify individuals at-risk for suicide attempts and death [2,55,56,57,58]. Moreover, assessing suicidal ideation with the PHQ-9 allowed for a direct comparison to recent reports of the frequency of suicidality in the legal profession [4]. Participants were considered to have endorsed suicidality if they selected “Several days”, “More than half the days”, or “Nearly every day” to the item “How often have you had thoughts that you would be better off dead, or of hurting yourself”. Participants who selected “Never” for this item were classified as not having suicidality. 

### 2.3. Data Analysis

Demographic and mental health severity scores on the PHQ-9 were compared between men and women using chi-square analyses. Logistic regression analyses were performed to identify associations between predictor variables (e.g., Work–Family Conflict, Work Overcommitment,) and the outcome variables (PHQ-9 suicidality) while controlling for covariates (e.g., COVID-19 impact on PHQ-9 items). 

Predictors were entered one at a time in a stepwise fashion, and their impact on the model’s overall fit was assessed. Those that significantly contributed to the model were entered into the primary study model. A sensitivity analysis was then conducted to examine the impact of COVID-19 on the primary model by entering a variable representing COVID-19 impact on PHQ-9 suicidality (e.g., a single item added at the end of assessments that asked whether problems defined in the PHQ-9 increased, decreased, or stayed the same since COVID-19). P-values for multiple comparisons were corrected using Holm–Bonferroni adjustments.

## 3. Results

Of the 80,000 members of the CLA and D.C. Bar that were randomly selected and received a study invitation, 5292 consented. The sample was restricted to lawyers who were employed part- or full-time in a legal setting at the time of the survey and who had complete data on the study measures. The final sample consisted of 1962 participants.

### 3.1. Descriptive Results

#### 3.1.1. Frequency of Suicidal Ideation

Approximately 8.5% (N = 165) of the participants reported thoughts they would be better off dead, or of hurting themselves “Several days”, “More than half the days”, or “Nearly every day” and were grouped in the suicidal ideation group. The remaining 91.5% (N = 1797) selected “Not at all” for PHQ-9 item 9 and were grouped in the non-suicidal ideation group. 

#### 3.1.2. Demographic Variables

Groups were compared on demographic, occupation, and mental health variables prior to model testing. Women comprised approximately 51% (N = 991) of the sample. Table 1 shows the distribution of demographic variables for participants who endorsed PHQ-9 suicidality vs. those who did not (“Not at all”). There were no differences in the proportion of men and women who endorsed suicidality as a function of gender or race. However, with respect to age, lawyers who endorsed (vs. did not endorse) suicidality tended to be younger. For example, a significantly greater proportion of lawyers from the suicidality group (compared to the non-suicidality group) belonged to the two youngest age groups (30 or younger and 31–40) and a lower proportion of suicidality endorsers belonged to the oldest age group (61 or older).

#### 3.1.3. Work-Related Demographics

Work-related sample demographics are shown in Table 2 for both groups. The total number of hours worked in a week, the participant’s law practice setting, and whether the participant’s legal position involved litigation did not significantly differ between groups. There was a trend that approached but did not reach significance (*p* = 0.051) with regards to position in the legal profession, such that a greater proportion of lawyers in the most junior level (junior associate) endorsed (vs. did not endorse) suicidality. 

#### 3.1.4. Mental health Diagnoses and Symptom Severity

There were no significant group differences concerning current drinking status (current drinker, former drinker, or lifetime abstainer). However, regarding substance use status, a significantly greater proportion of endorsers of suicidality identified as a current substance user (data not shown). Table 3 shows the proportions of lawyers in each suicidality group with a past 12-month mental health diagnosis and the proportion within the severity classifications of the PHQ-9, AUDIT-C, DAST, PSS, and the UCLA loneliness scale. Overall, a greater proportion of lawyers who endorsed suicidal ideation had a current mental health condition (Depression, Anxiety, PTSD, Bipolar Disorder, AUD, or other) and were significantly more likely to be in the moderate, moderately severe, or severe range of depression as measured by the PHQ-9. Similar results indicating greater severity among the suicidality vs. the non-suicidality group were reported concerning (1) hazardous drinking (AUDIT-C), (2) substance use severity (DAST), (3) moderate to high stress (PSS), and (4) loneliness (UCLA Loneliness Scale). 

Table 4 shows the proportion of participants in each group with responses to survey items assessing whether participants believed their time in the legal profession has been detrimental to their mental health, led to increased alcohol/substance use, or caused them to contemplate leaving the profession due to mental health, burnout, or stress. A significantly greater proportion of lawyers in the suicidality group reported that their time in the legal profession was detrimental to their mental health, caused an increase in their substance/alcohol use, and considered leaving the profession due to mental health problems or burnout. 

### 3.2. Predictors of Suicidal Ideation

The results of the logistic regression analyses examining predictors of endorsement of suicidal ideation among lawyers are shown in Table 5. The following predictors did not significantly contribute to the model: alcohol and substance use severity, age, and work-family conflict. As a result, these items were removed in the final, simplified model. The final model contained the following predictors: gender, history of a mental health diagnosis, loneliness, perceived stress, and work overcommitment. Results of the model indicated that the odds of having suicidal ideation were 2.2 times higher among lawyers with high work overcommitment and 1.6 times higher among lawyers with an intermediate level of work overcommitment. Lawyers who screened as lonely on the UCLA loneliness scale were 2.8 times more likely to endorse suicidality than lawyers who did not screen as lonely. Gender was also significantly associated with suicidality, with men being 2 times more likely to endorse suicidality compared to women. Lawyers with a history of at least one mental illness diagnosis were 1.8 times more likely to endorse suicidality compared to lawyers with no history of mental illness. Finally, compared to lawyers with low perceived stress, those with high or intermediate stress levels were 22 times more likely and 5.5 times more likely, respectively, to endorse suicidality. 

### 3.3. Sensitivity Analysis

Accounting for COVID-19. It is important to acknowledge that data collection for the study occurred during the COVID-19 pandemic. In an attempt to control the pandemic’s collateral burden on the study outcomes, responses to a single item assessing whether participants believed their PHQ-9 depression symptoms changed since the beginning of the pandemic was entered into the model as a covariate (“Thinking back to before the COVID-19 pandemic, do you believe the frequency of these problems has remained the same, decreased, or increased?”). The results of the model indicated that the perceived influence of COVID-19 on PHQ-9 responses was not a significant predictor of suicidality and that the ORs and significance levels of all the predictors noted in Table 5 were maintained (Supplement Table S1). 

## 4. Discussion

Given the disproportionately high rates of lawyers who contemplate suicide, this study was designed to identify risks for suicidal ideation in the legal profession. To the best of our knowledge, this is the first study to report on factors related to suicidal ideation among lawyers randomly selected from a large sample of practicing lawyers. The first, most notable finding was that 8.5% of lawyers in our sample endorsed suicidal ideation as assessed by the PHQ-9, which is twice as high as the rate in in the general working population and closer to the rate among Utah lawyers (11.9%) noted by Thiese et al. (2021) [4]. The high prevalence of suicidal ideation among lawyers warrants further attention and mitigation efforts that address associated risk factors. 

In addition to the high overall rate of suicidal ideation among lawyers, our study demonstrated that perceived stress was significantly associated with increased risk for suicidal thoughts. In fact, the odds of contemplating suicide were a remarkable 22 times higher among lawyers with high (vs. low) stress on the PSS. This finding supports prior studies indicating that perceived stress (as assessed by the PSS) predicts suicidal ideation and suicidal behavior in other populations [19,59,60]. However, the highly conspicuous extent to which it relates to lawyer suicide risk specifically would suggest that stress should be a primary target of suicide prevention and mitigation strategies for that population. A twofold strategy whereby stressors in lawyers’ lives are reduced, and their stress tolerance is enhanced, would seem to be the most efficacious approach for mitigating the stress-suicidality risk. To date, however, most efforts to reduce stress within the legal profession have tended to target the individual, e.g., through the provision of personal stress management tools and self-care resources. Where employers have attempted to address the more structural and systemic precipitators of stress (i.e., unrealistic time pressures, unclear expectations, workload control, lack of feedback), employees have generally rated their efforts as ‘highly ineffective’ [5]. Simply put, it would seem the legal profession has been better at alleviating the effects of stress than in throttling the causes. 

To be clear, interventions aimed at helping individuals better cope with stress should remain an essential element of any legal employer’s efforts to improve lawyer mental health. Evidence-based self-care interventions for coping with perceived stress have been demonstrated to be effective in numerous settings [61,62,63]. Considering the profound impact of stress on lawyer suicidality, we believe that all options should remain viable for mitigating stress, including the examination and recalibration of organizational or profession-wide attitudes, norms, and cultures relating to work. Placing increased onus for change on the systems and structure of the profession, as opposed to individual lawyers, would seem appropriate due to the reported experiences of lawyers themselves. Specifically, a significantly greater proportion of lawyers who contemplated suicide indicated that working in the legal profession was detrimental to their mental health and contributed to their substance use, and feelings of burnout (See Table 4). Furthermore, such systemic introspection is both needed and timely in the wake of the COVID-19 pandemic. As noted in a recently published report on workplace mental health from the U.S. Surgeon General, organizational leaders, managers, supervisors, and workers alike have an unprecedented opportunity to examine the role of work in our lives and explore ways to better enable thriving in the workplace and beyond. 

The importance of individual and organizational solutions for creating more mentally healthy workplaces is well-established in the literature [64], with upstream approaches being proposed as the most effective to prevent suicide and workplaces being ideal contexts to apply such approaches [65]. By seeking to reduce the incidence and impact of perceived stress among their lawyers, legal employers could be going far upstream with the potential for meaningful reductions in suicidal ideation. An obvious but important fact must be noted, namely that stressors outside of work could certainly contribute to lawyer suicidal ideation and therefore escape the reach of an employer’s efforts to reduce stress. To speak practically, employers have an outsized role to play after numerous surveys and studies confirm that occupational pressures and fears are exceedingly the leading source of stress for American adults [66]. 

Social isolation or loneliness is noted as a common experience among lawyers and law students, often related to the demanding and high-stress nature of the legal profession, as well as the competitive and individualistic culture of law firms and law schools [15,16]. In the present study, lawyers experiencing high levels of loneliness were nearly three times as likely to experience suicidal ideation as those experiencing low levels of loneliness. This finding aligns with previous work demonstrating a relationship between loneliness and suicide risk [18,20,22,23]. Importantly, research has also shown that a sense of relatedness, i.e., how you connect, or relate to others, and whether you feel a sense of belonging at work, among lawyers strongly correlates with improved wellbeing [67]. By making collaboration and regular social interactions in the work environment more of a priority, employers may be able to help mitigate some of the loneliness their lawyers experience. Any such efforts will undoubtedly be complicated by remote and hybrid working models that now predominate the legal field, especially as recent reports from the field suggest that many lawyers are reluctant to return to the office [68]. Given the high rates of alcohol misuse among lawyers and the strong connection between workplace permissiveness towards alcohol and the risk of hazardous drinking among lawyers [7], efforts to combat loneliness and isolation should avoid reliance on alcohol-based events as a primary means of increasing socialization and connection. 

Turning to gender, the odds of suicidal ideation were two times higher for men than women. This surprising finding stands in contrast to the ‘gender paradox of suicidal behavior’ demonstrated by other research, whereby it has been shown that women in most Western countries have higher rates of suicidal ideation but lower rates of mortality than men [69,70]. This finding is also notable because women attorneys experienced higher levels of depression, anxiety, and hazardous drinking than men, which would typically suggest a higher level of corresponding suicide risk. However, after controlling for these variables in our final model, it was revealed that men were more likely to experience suicidal ideation. This would suggest that factors not included in our model, and which may not typically be tied to suicidality, are affecting the tendency of male attorneys to experience suicidal ideation. Further research would be needed to determine the specific reasons for the higher rates of suicidal ideation among male lawyers and the apparent inapplicability of the gender paradox of suicidal behavior to the lawyer population.

Relating to work overcommitment, lawyers with high (vs. low) levels of work overcommitment were two times as likely to endorse suicidal ideation, while those with intermediate levels of overcommitment were 1.5 times more likely to report such thoughts. Work overcommitment, as measured by the ERI questionnaire, has been described as an intrinsic or personality-based coping factor which reflects the need for approval, esteem, and control and it has been shown to be significantly associated with cynicism, exhaustion, and greater psychological distress [71]. According to the ERI model proposed by Siegrist and Montano, 2014 [53], overcommitment involves a desire to control one’s work environment and an inability to disconnect from work. Evidence of work overcommitment includes thinking about work immediately upon waking, having people tell you that you sacrifice too much for work, and an inability to relax and switch off work, among other things. High levels of overcommitment to work have been shown to play a detrimental role in lawyer mental health [72], but interventions aimed at reducing such work overcommitment face an uphill climb in the legal profession. Being overly dedicated to one’s work is generally highly rewarded in law, beginning in law school and continuing throughout many legal work environments where lawyers are often promoted based on their observed level of commitment to their work, their firm, and their clients. At the same time, research has shown that extrinsic validations and rewards (i.e., grades, rankings, honors, and financial rewards) do not predict lawyer wellbeing but instead that these external considerations that often dominate law schools and law practice are of subordinate importance to lawyer happiness when compared to other basic psychological needs, such as autonomy, relatedness to others, and competence [66]. By raising awareness of the notable downsides of being too committed to one’s work, encouraging lawyers to set and maintain appropriate boundaries in their lives, and reframing notions of success to prioritize intrinsic over extrinsic rewards, stakeholders in the legal profession may be able to temper or modulate the harmful effects of work overcommitment without asking lawyers to fully abandon the dedication to their work that may have greatly contributed to elements of their prior success and achievements.

Findings from the present study are consistent with previous research linking mental health disorders (e.g., depression, anxiety) to increased risk for suicidal ideation [73,74]. For example, while suicide accounts for about 1.4% of deaths worldwide, it has been estimated that the risk climbs to 5–8% for those with a mental disorder, such as depression, alcoholism, and schizophrenia [75]. It is well established that mental health disorders can disrupt cognitive and emotional functioning, leading to negative thoughts and behaviors, including suicidal ideation [73]. The present study adds to this literature by demonstrating that these factors are also relevant in the specific context of the legal profession because lawyers with a prior mental health diagnosis were nearly twice as likely to demonstrate suicidal ideation. 

Another possible explanation of heightened suicidal ideation among lawyers is workplace culture which may promote unhealthy coping mechanisms and discourage seeking help for mental health problems. Previous research has demonstrated a pronounced reluctance on the part of lawyers to disclose or seek help for a mental health disorder, often due to fear of negative career or professional repercussions [3]. This “sink or swim” mentality and stigma surrounding seeking help for mental health problems may create a toxic work environment that contributes to the high rates of suicidal ideation in the legal profession. One strategy to address this issue involves destigmatizing mental health problems and promoting a culture of help-seeking within the legal profession when mental health problems arise. 

Previous research indicates work–family conflict, alcohol use (AUDIT-C), and drug use (DAST) are associated with suicide risk. However, they were not associated with contemplating suicide among our sample of lawyers. This could be due to an overlap between these factors and perceived stress or other variables in the model. For example, other research demonstrates that scores on the AUDIT-C and DAST strongly correlate with perceived stress [76]. As such, it is possible that due to the overlap and strong relationship between perceived stress, alcohol use disorder, and substance use disorder, that the predictors of AUDIT-C and DAST scores did not emerge as significant while perceived stress did. It is important to emphasize that several lines of research implicate alcohol and substance use with suicidality, while several other lines of research demonstrate that lawyers engage in hazardous levels of alcohol and substance use at rates much higher than the general population. Although risky drinking was not a significant predictor of suicidality in this study (likely for the reasons cited above), ours and other’s past work clearly indicates a strong connection between problem drinking and psychological distress among lawyers. It is therefore possible that problem drinking impacts the risk for suicidal ideation among lawyers indirectly, by contributing to elements of psychological distress (e.g., perceived stress, poor mental health). Considering these findings, more research is needed to examine the specific contribution of risky drinking to suicidality among lawyers and it would be inappropriate to conclude that it does not meaningfully contribute to their suicide risk.

## 5. Limitations

There are limitations to the present study that should be considered when interpreting the results. First, the cross-sectional design of the study means that causality cannot be inferred. It is possible that suicidal ideation may also be a cause rather than just a consequence of the predictor variables. Longitudinal studies are needed to establish the direction of the relationship between these variables.

Second, the sample of lawyers in the present study was drawn from two jurisdictions only, California and Washington, D.C. Although those jurisdictions have among the largest lawyer populations in the United States and thereby provide for a large and diverse sample, they may not be representative of the legal profession as a whole. Further research would help confirm the generalizability of these findings to other geographic regions.

Third, the present study relied on self-report measures to assess predictor and outcome variables. Self-report measures are susceptible to bias and may not always reflect an individual’s true thoughts, feelings, or behaviors. Future research using objective measures (e.g., medical records, performance assessments) may provide a clearer picture of the relationship between these variables, though such research may be difficult or impractical to conduct. 

Finally, although AUDIT scores did not predict suicidal ideation in the present study, drinking is still very relevant to the discussion of suicide in this population given the high rates of problem drinking among lawyers [3,7] and the well-established connection between substance misuse and suicide generally [77]. Future research should continue to examine the relationship between alcohol use and suicidal ideation in this population.

## 6. Conclusions

Efforts are underway within the legal profession to improve mental health, reduce the stigma associated with mental health disorders, and increase the overall wellbeing of lawyers. To support and inform those efforts, an enhanced empirical understanding of the profession’s unique mental health risks is essential, including a better understanding of why lawyers are much more likely than the average person to experience suicidal thoughts. This research has begun to answer that question. To summarize, our findings suggest the profile of a lawyer with the highest risk for suicide is a lonely or socially isolated male with a high level of unmanageable stress, who is overly committed to their work, and may have a history of mental health problems. The heightened risk of suicidal ideation extends well beyond this specific profile, however, thereby necessitating a sustained focus on the factors we identified as predictive of that risk. Overall, these findings underscore the need for interventions to address work-related stress and loneliness in the legal profession. This may include providing education, resources, and support for lawyers to better manage their workload, modifying work demands and expectations, and promoting a culture of openness and support within law firms. Additionally, targeting interventions towards male lawyers may be particularly important given their higher risk of suicidal ideation. Further research is needed to continue exploring the dynamics of the relationship between work overcommitment, loneliness, perceived stress, and suicidal ideation in this population.

## Figures and Tables

**Table 1:** Demographics according to endorsement of PHQ-9 suicidal ideation (N = 1962).

	No Suicidal Ideation (N = 1797)		Suicidal Ideation (N = 165)		ꭓ^2^, *p*-Value
	N	%	N	%	
** Gender **					ꭓ ^ 2 ^ (1) = 1.064, 0.302
Female	914	92.2%	77	7.8%	
Male	883	90.9%	88	9.1%	
** Age **					ꭓ ^ 2 ^ (4) = 18.81, <0.001
30 or younger	126 ^ a ^	85.7%	21 ^ b ^	14.3%	
31–40	465 ^ a ^	89.4%	55 ^ b ^	10.6%	
41–50	425 ^ a ^	93.2%	31 ^ a ^	6.8%	
51–60	408 ^ a ^	91.1%	40 ^ a ^	8.9%	
61 or older	373 ^ a ^	95.4%	18 ^ b ^	4.6%	
** Race **					ꭓ ^ 2 ^ (6) = 10.04, 0.123
Asian or Pacific Islander	125	86.8%	19	13.2%	
Black/African American	85	90.4%	9	9.6%	
Caucasian/White	1457	92.3%	122	7.7%	
Latino/Hispanic	62	91.2%	6	8.8%	
Native American	3	100.0%	0	0.0%	
More than one race	40	83.3%	8	16.7%	
Other	16	94.1%	1	5.9%	

Within each row, each superscript letter denotes column proportions that did not differ significantly at the 0.05 level according to Pearson Chi-Square tests.

**Table 2:** Work-related demographics of the study sample according to endorsement of PHQ-9 suicidal ideation (N = 1962).

	No Suicidal Ideation (N = 1797)		Suicidal Ideation (N = 165)		ꭓ^2^, *p*-Value
	N	%	N	%	
**Hours worked in a typical week**					ꭓ^2^(7) = 9.674, *p* = 0.208
Less than 10 h	28	90.3%	3	9.7%	
11 to 20 h	65	98.5%	1	1.5%	
21 to 30 h	82	91.1%	8	8.9%	
31 to 40 h	405	91.6%	37	8.4%	
41 to 50 h	759	92.1%	65	7.9%	
51 to 60 h	348	90.9%	35	9.1%	
61 to 70 h	81	85.3%	14	14.7%	
71 h or more	25	92.6%	2	7.4%	
**Position in Legal Profession**					ꭓ^2^(6) = 14.021, *p* = 0.051
Managing partner	315	92.6%	25	7.4%	
Senior partner	262	93.9%	17	6.1%	
Junior partner	115	92.0%	10	8.0%	
Of counsel	138	91.4%	13	8.6%	
Senior associate	254	93.0%	19	7.0%	
Junior associate	195	85.9%	32	14.1%	
Other	414	91.9%	41	9.0%	
**Law Practice Setting**					ꭓ^2^(7) = 12.200, *p* = 0.094
Sole Practitioner—Private Practice	269	93.4%	19	6.6%	
Private Firm	740	90.7%	76	9.3%	
In-house lawyer: government, public interest, or nonprofit	445	92.5%	36	7.5%	
In-house lawyer: corporation or for-profit institution	233	91.7%	21	8.3%	
Judicial chambers (judge/hearing officer/clerk)	3	60.0%	2	40.0%	
Other law practice setting	39	86.7%	6	13.3%	
College or law school	6	85.7%	1	14.3%	
Other setting (not law practice)	15	100.0%	0	0.0%	
**Position Involves Litigation (Yes)**	1072	59.7%	105	63.6%	ꭓ^2^(1) = 1.393, *p* = 0.238

**Table 3:** The prevalence of mental health diagnoses, severity of depression, alcohol use, substance use, and loneliness in the study sample according to endorsement of PHQ-9 suicidal ideation (N = 1962).

	No Suicidal Ideation (N = 1797)		Suicidal Ideation (N = 165)		ꭓ^2^, *p*-Value
	N	%	N	%	
**Past 12-Month Mental Health Diagnosis**					
Depression					ꭓ^2^(2) = 132.47, *p* < 0.001
current	152 ^a^	9.7%	62 ^b^	41.6%	
lifetime	321 ^a^	20.5%	31 ^a^	20.8%	
no history	1096 ^a^	69.9%	56 ^b^	37.6%	
total	1569		149		
Anxiety Disorder					ꭓ^2^(2) = 65.033, *p* < 0.001
current	226 ^a^	14.5%	54 ^b^	39.7%	
lifetime	232 ^a^	14.9%	26 ^a^	19.1%	
no history	1096 ^a^	70.5%	56 ^b^	41.2%	
total	1554		136		
PTSD					ꭓ^2^(2) = 58.780, *p* < 0.001
current	22 ^a^	1.9%	12 ^b^	15.8%	
lifetime	54 ^a^	4.6%	8 ^b^	10.5%	
no history	1096 ^a^	93.5%	56 ^b^	73.7%	
total	1172		76		
Bipolar Disorder					ꭓ^2^(2) = 17.852, *p* < 0.001
current	3 ^a^	0.3%	2 ^b^	3.3%	
lifetime	12 ^a^	1.1%	2 ^a^	3.3%	
no history	1096 ^a^	98.6%	56 ^b^	93.3%	
total	1111		60		
Alcohol Use Disorder					ꭓ^2^(2) = 13.739, *p* < 0.001
current	8 ^a^	0.7%	3 ^b^	4.8%	
lifetime	31 ^a^	2.7%	4 ^a^	6.3%	
no history	1096 ^a^	96.3%	56 ^b^	88.9%	
total	1135		63		
Substance Use Disorder					ꭓ^2^(2) = 2.712, *p* = 0.258
current	4	0.4%	1	1.7%	
lifetime	11	1.0%	1	1.7%	
no history	1096	98.6%	56	96.6%	
total	1111		58		
Other					ꭓ^2^(2) = 17.852, *p* < 0.001
current	14 ^a^	1.2%	5 ^b^	7.9%	
lifetime	20 ^a^	1.8%	2 ^a^	3.2%	
no history	1096 ^a^	97.0%	56 ^b^	88.9%	
total	1130		63		
**PHQ-9-Depression Severity**					ꭓ^2^(4) = 541.079, *p* < 0.001
None/Minimal	1011 ^a^	57.8%	12 ^b^	7.4%	
Mild	517 ^a^	29.5%	33 ^b^	20.4%	
Moderate	183 ^a^	10.5%	51 ^b^	31.4%	
Moderately Severe	34 ^a^	1.9%	46 ^b^	28.4%	
Severe	5 ^a^	0.30%	20 ^b^	12.3%	
**AUDIT-C-Alcohol Use Severity**					ꭓ^2^(2) = 7.881, *p* < 0.05
Low risk	892 ^a^	49.6%	74 ^a^	44.8%	
Risky drinking	389 ^a^	21.6%	27 ^a^	16.4%	
Hazardous drinking	516 ^a^	28.7%	64 ^b^	38.8%	
**DAST-Substance Use Severity**					ꭓ^2^(3) = 24.952, *p* < 0.001
Lifetime abstinence	1418 ^a^	78.9%	119 ^b^	72.1%	
No problems reported	90 ^a^	5.0%	6 ^a^	3.6%	
Low	251 ^a^	14.0%	26 ^a^	15.8%	
Moderate to severe	38 ^a^	2.1%	14 ^b^	8.5%	
**PSS—Perceived Stress Scale**					ꭓ^2^(2) = 237.645, *p* < 0.001
Low	812 ^a^	45.2%	10 ^b^	6.1%	
Moderate	897 ^a^	49.9%	98 ^b^	59.4%	
High	88 ^a^	4.9%	57 ^b^	34.5%	
**UCLA Loneliness Scale**					ꭓ^2^(1) = 110.338, *p* < 0.001
Not Lonely	1224 ^a^	68.1%	45 ^b^	27.3%	
Lonely	573 ^a^	31.9%	120 ^b^	72.7%	

Each subscript letter denotes a subset of whose column proportions do not differ significantly from each other at the 0.05 level.

**Table 4:** Proportion of participants with and without PHQ-9 suicidal ideation with responses to items reflecting the perceived relationship between personal mental health and time in the legal profession (N = 1962).

	No Suicidal Ideation (N = 1797)		Suicidal Ideation (N = 165)		ꭓ^2^, *p*-Value
	N	%	N	%	
**Has your time in the legal profession been detrimental to your mental health?**					ꭓ^2^(2) = 110.436, *p* < 0.001
yes	476 ^a^	27.1%	106 ^b^	66.3%	
no	943 ^a^	53.8%	30 ^b^	18.8%	
unsure	335 ^a^	19.1%	24 ^a^	15.0%	
**Has your time in the legal profession caused your use of alcohol and/or other drugs to increase?**					ꭓ^2^(2) = 50.771, *p* < 0.001
yes	248 ^a^	14.1%	55 ^b^	34.2%	
no	1385 ^a^	78.9%	89 ^b^	55.3%	
unsure	122 ^a^	7.0%	17 ^a^	10.6%	
**Are you considering, or have you left the profession due to mental health, burnout or stress?**					ꭓ^2^(2) = 81.932, *p* < 0.001
yes	320 ^a^	18.2%	74 ^b^	46.0%	
no	1352 ^a^	77.0%	72 ^b^	44.7%	
unsure	83 ^a^	4.7%	15 ^b^	9.3%	

Each subscript letter denotes a subset of whose column proportions do not differ significantly from each other at the 0.05 level.

**Table 5:** Predictors of PHQ-9 suicidal ideation among lawyers (N = 1962).

	OR	95% CI
Gender (ref. female)		
Male	2.005 ***	(1.401–2.870)
Dx History (ref. no Dx history)		
Yes	1.822 ***	(1.26–2.63)
UCLA Loneliness		
Lonely	2.793 ***	(1.90–4.103)
PSS-Perceived Stress Scale (ref. Low)		
Low		
Intermediate	5.475 ***	(2.750–10.90)
High	22.392 ***	(10.30–48.64)
Work Overcommitment (ref. Low)		
Low		
Intermediate	1.585	(.850–2.96)
High	2.207 **	(1.206–4.039)

* significant difference from referent (** *p* ≤ 0.01; *** *p* ≤ 0.001); *OR* = odds ratio; *CI* = confidence interval.

## Data Availability

Data cannot be shared publicly because they involve human research participants and contain potentially sensitive information related to mental health and substance use. Researchers who meet the criteria for access to confidential data may request to access the data by contacting the corresponding author and completing a University of Minnesota Data Use Agreement.

## Supplementary Materials

The following supporting information can be downloaded at: https://www.mdpi.com/article/10.3390/healthcare11040536/s1, Table S1: Predictors of PHQ-9 suicidal ideation among lawyers controlling for perceived influence of COVID-19 on PHQ-9 depression symptoms (N = 1962).
