# Supplementary material for: Stressed, Lonely, and Overcommitted: Predictors of Lawyer Suicide Risk
Source: Healthcare (Basel). 2023 Feb 11;11(4):536. doi: 10.3390/healthcare11040536 (PMC9956925; doi:10.3390/healthcare11040536)
Supplement: Supplementary file 1 [file healthcare-11-00536-s001.zip › healthcare-2181911-supplementary.pdf]

Supplementary Table S1. Predictors of PHQ-9 suicidal ideation among lawyers controlling for perceived influence of COVID-19 on PHQ-9 depression symptoms (N = 1962).

	OR	95% CI
**Gender** (ref. female)		
Male	1.960 ***	(1.367–2.811)
**Dx History** (ref. no hx)		
Yes	1.804 ***	(1.247–2.63)
**UCLA Loneliness** (ref. not Lonely)		
Lonely	2.842 ***	(1.932–4.180)
**PSS-Perceived Stress Scale** (ref. Low)		
Low		
Intermediate	5.960 ***	(2.959 -12.004)
High	24.805 ***	(11.221–54.833)
**Work Overcommitment** (ref. Low)		
Low		
Intermediate	1.558	(0.835–2.906)
High	2.150 **	(1.175–3.935)
**Perceived COVID-19 Impact on PHQ-9 depression** (ref. Remained the same)		
Remained the same		
Decreased	0.495	(0.182–1.351)
Increased	0.695	(0.472–1.023)

* Significant difference from referent ** $p \leq 0.01$; *** $p \leq 0.001$); *OR* = odds ratio; *CI* = confidence interval.
